# Temporary blood pressure drop after bevacizumab administration is associated with clinical course of advanced colorectal cancer

**DOI:** 10.1038/bjc.2011.398

**Published:** 2011-10-27

**Authors:** M Kanai, H Ishiguro, Y Mori, T Kitano, T Nishimura, S Matsumoto, K Yanagihara, T Chiba, M Toi

**Affiliations:** 1 Outpatient Oncology Unit, Kyoto University Hospital, 54 Shogin-Kawahara-cho, Sakyo-ku, Kyoto 606-8507, Japan; 2 Department of Gastroenterology and Hepatology, Kyoto University Hospital, Sakyo-ku, Kyoto 606-8507, Japan; 3 Department of Breast Surgery, Kyoto University Hospital, Sakyo-ku, Kyoto 606-8507, Japan

**Keywords:** bevacizumab, blood pressure, hypotension, predictive marker

## Abstract

**Background:**

A blood pressure drop after bevacizumab administration and its clinical significance have not been previously reported.

**Methods:**

Blood pressure data at 0, 90, and 180 min after a total of 162 bevacizumab administrations in 81 advanced colorectal cancer patients were retrospectively investigated.

**Results:**

Twenty-five patients (30%) demonstrated an average temporary drop of 20 mm Hg or more in systolic blood pressure. We classified these 25 patients as group A and the others as group B. Median time-to-treatment failure (TTF) was significantly longer in group A than in group B (291 *vs* 162 days; *P*=0.02). Furthermore, the proportion of patients who required intervention with antihypertensive drugs during bevacizumab treatment was significantly higher in group A than in group B (36% *vs* 4% *P*<0.01).

**Conclusion:**

This study suggests that a temporary blood pressure drop after bevacizumab administration could be a predictive marker for bevacizumab treatment.

Bevacizumab is a recombinant humanised monoclonal antibody that binds to vascular endothelial growth factor (VEGF) A and is now widely used for the treatment of colorectal cancer in combination with other cytotoxic drugs (Hurwitz *et al*, 2004; Giantonio *et al*, 2007). Hypertension is one of the most common adverse effects and its total incidence is reported to be 10–30% (Zhu *et al*, 2007). Therefore, active monitoring of blood pressure is recommended in daily clinical practice during bevacizumab treatment (Maitland *et al*, 2010). In the course of this blood pressure monitoring, we have observed that some patients experience a temporary blood pressure drop after administration of bevacizumab. Moreover, among these patients, we noted one who responded to the third-line therapy using bevacizumab after failure of oxaliplatin and irinotecan. These observations prompted us to perform the current study to investigate the incidence of a drop in blood pressure after bevacizumab administration, and its association with the clinical course of patients with advanced colorectal cancer.

## Patients and methods

### Patients

Between August 2007 and October 2010, 85 patients with colorectal cancer underwent palliative chemotherapy using bevacizumab at Kyoto University Hospital. For these patients, we obtained data using both the designated database system (CyberOncology, CyberLaboratory Co. Ltd., Ibaragi, Japan) (Matsumoto *et al*, 2007) and the hospital’s electronic medical records system on blood pressure values at 0 min (before administration), at 90 min and 180 min after the initiation of bevacizumab during the first three cycles of bevacizumab treatment. We also investigated the status of antihypertensive drug intake before and after bevacizumab treatment. Out of the 85 patients, there were 81 for whom two or more sets of blood pressure data from the first three cycles were available. Therefore, we retrieved data from a total of 162 bevacizumab administrations in 81 patients (data from two separate administrations per patient). The blood pressure value at 180 min was not monitored in three patients who received capecitabine/bevacizumab, due to the short infusion period of this regimen.

### Treatment with bevacizumab

Bevacizumab was administered in combination with one of the following regimens (fluorouracil/leucovorin/irinotecan (FOLFIRI), fluorouracil/leucovorin/oxaliplatin (mFOLFOX6), fluorouracil/leucovorin (sLV5FU2), capecitabine/oxaliplatin (XELOX), or capecitabine) as previously reported (Kabbinavar *et al*, 2003; Fuchs *et al*, 2007; Saltz *et al*, 2008). None of the patients received bevacizumab as monotherapy. The first/second/third dose of bevacizumab was infused over 90/60/30 min, respectively, as previously reported (Margolin *et al*, 2001).

### Statistical methods

Baseline patient characteristics were compared using the *χ*^2^-test or Fisher’s exact test for dichotomous variables or the Mann–Whitney *U*-test for continuous variables. Time-to-treatment failure (TTF) was defined as the interval between the date of initiation and discontinuation of treatment and estimated by the Kaplan–Meier method. Patients not experiencing an event were censored at the last follow-up visit. Comparisons of the time-to-event distributions were made using the log-rank test, with the hazard ratio (HR) and its 95% confidence intervals (CIs) calculated from a Cox regression model. All *P*-values are two-sided. All statistical analyses were performed using SPSS version 11.0J (SPSS Japan, Tokyo, Japan).

## Results

### Patient characteristics

Patient baseline characteristics are summarised in Table 1. Sixteen patients (20%) had received antihypertensive drugs before the first date of bevacizumab treatment and eleven patients (14%) required intervention with antihypertensive drugs during bevacizumab treatment.

### Blood pressure changes after bevacizumab administration

**Figure 1:**
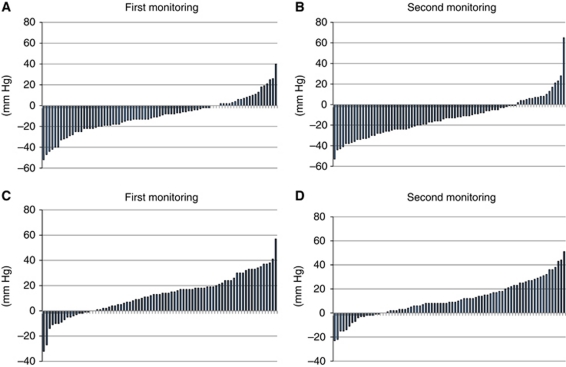
Waterfall plot analysis of systolic blood pressure changes after bevacizumab administration: (**A**) first monitoring between 0 and 90 min, (**B**) second monitoring between 0 and 90 min, (**C**) first monitoring between 90 and 180 min, and (**D**) second monitoring between 90 and 180 min.

**Figure 2:**
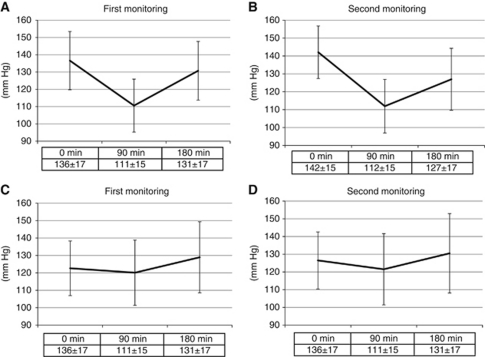
Systolic blood pressure values (mean±s.d.) at 0, 90, and 180 min after bevacizumab administration: (**A**) first monitoring from group A, (**B**) second monitoring from group A, (**C**) first monitoring from group B, and (**D**) second monitoring from group B.

Figure 1 shows a waterfall plot analysis of the change in systolic blood pressure. The proportion of patients experiencing a blood pressure drop was greater than those showing a blood pressure elevation between 0 and 90 min (Figure 1A and B), and this proportion was reversed between 90 and 180 min (Figure 1C and D). A total of 25 patients (30%) demonstrated a temporary drop of 20 mm Hg or more on average and we classified these patients as group A and the others as group B. Patient characteristics of group A and group B are summarised in Table 1, and there were no significant differences between the two groups in their baseline characteristics. Systolic blood pressure (mean±s.d.) at 0, 90, and 180 min is shown in Figure 2. In group A, the mean blood pressure drop between 0 and 90 min was 25 and 30 mm Hg in the first and second monitoring, respectively, compared with 3 and 5 mm Hg in group B. Thus, similar trends were observed through two separate cycles in each group.

### Association between temporary blood pressure drop and TTF

**Figure 3:**
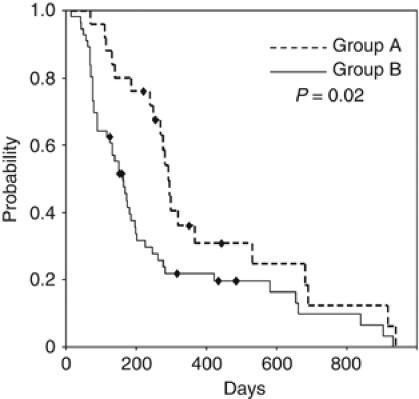
Median TTF of patients experiencing a temporary blood pressure drop of 20 mm Hg or more (group A) and the others (group B).

We then compared TTF between group A and group B. TTF was significantly longer in group A (291 days; 95% CI, 266–316 days) compared with group B (162 days; 95% CI, 122–202 days) with an HR of 0.53 (95% CI, 0.31–0.89; *P*=0.02) (Figure 3).

## Discussion

Since hypertension associated with bevacizumab treatment has been well documented (Hurwitz *et al*, 2004; Zhu *et al*, 2007), many physicians pay attention to this phenomenon, whereas a blood pressure drop is likely to be overlooked. In this study, 25 patients (30%) experienced a temporary systolic blood pressure drop of 20 mm Hg or more after bevacizumab administration. We consider this observation not to be a result of chance, physiological variability or inaccurate measurement for the following reasons.

First, the drop in blood pressure was a temporary phenomenon and blood pressure returned to baseline levels between 90 and 180 min after bevacizumab administration (Figures 1 and 2). If this drop was the result of a resolution of prior blood pressure elevation due to the white-coat effect or pre-injection anxiety, then the reduced blood pressure at 90 min would be more likely to remain at this lower level until 180 min.

Second, this phenomenon was observed through two separate cycles.

Third, in the first cycle bevacizumab was infused over 90 min and no other drugs were infused until 90 min.

Out of the 25 patients classified as group A, 10 received anti-epidermal growth factor receptor monoclonal antibody and blood pressure was monitored at 0 and 60 min after the administration of this antibody during the first cycle. Mean change in systolic blood pressure was 3 mm Hg, and no patients demonstrated a blood pressure drop of 20 mm Hg or more. These results also support the idea that a blood pressure drop after bevacizumab administration was not merely caused by a result of chance, physiological variability or inaccuracy of blood pressure measurement.

In line with our current results, Iqbal *et al* (2007) reported the case of a patient with colorectal cancer who developed bevacizumab-associated hypotension. We speculate that more similar but unreported cases exist in daily clinical practice.

Next, we investigated the association between this phenomenon and TTF because recent studies have suggested a positive correlation between hypertension after bevacizumab treatment and clinical outcome (Scartozzi *et al*, 2009; Dahlberg *et al*, 2010; De Stefano *et al*, 2011). Even after adjustment for treatment line and regimen, the results show a significantly longer TTF in group A than in group B (*P*=0.02; Figure 3). Furthermore, the proportion of patients who required intervention with antihypertensive drugs during bevacizumab treatment was significantly higher in group A compared with group B (36% *vs* 4% *P*<0.01; Table 1), which supports previous studies demonstrating that bevacizumab-related hypertension was positively associated with clinical outcome. Our study is limited because it is a retrospective analysis of a small sample, including various treatment lines and regimens. However, a designated database system (CyberOncology) enables us to record TTF at the time an event occurs (Matsumoto *et al*, 2007), and we believe this system has contributed to improving the accuracy of the current data. In fact, TTF of patients who received bevacizumab as first-line or second-line treatment was 295 days (95% CI, 167–421 days) and 199 days (95% CI, 148–249 days), respectively. These results are comparable with previous data of progression-free survival from large prospective clinical trials evaluating the efficacy of bevacizumab in patients with advanced colorectal cancer (Hurwitz *et al*, 2005; Fuchs *et al*, 2007; Giantonio *et al*, 2007; Saltz *et al*, 2008).

In summary, our current study demonstrates that, in some patients, blood pressure drops temporarily after bevacizumab administration to varying degrees, and that this phenomenon is associated with a longer TTF. Since this phenomenon was observed from the first cycle of bevacizumab treatment, it could predict the clinical course of treatment at an earlier time point than bevacizumab-related hypertension. Future prospective studies with a larger cohort are warranted to verify the results of current study and to clarify its underlying mechanism.

## Figures and Tables

**Table 1:** Patient characteristics

**Characteristic**	**All patients (*n*=81)**	**Group A (*n*=25)**	**Group B (*n*=56)**	***P*-value**
*Gender—no. (%)*				
Male	42 (52)	9 (36)	33 (59)	0.09
Female	39 (48)	16 (64)	23 (48)	
				
*Age (years)*				
Median	64	64	65	0.93
Range	34–82	42–81	34–82	
				
*Primary tumour—no. (%)*				
Colon	51 (63)	15 (60)	36 (64)	0.71
Rectum	30 (37)	10 (40)	20 (36)	
				
*Metastatic site*				
Liver	48	11	37	
Lung	37	13	24	
Other	46	17	29	
				
*No of sites—no. (%)*				
One	42 (52)	12 (48)	30 (54)	0.77
More than one	39 (48)	13 (52)	26 (47)	
				
*CEA* *—no. (%)*				
<10 ng ml^−1^	27 (33)	8 (32)	19 (34)	0.93
≧10 ng ml^−1^	54 (67)	17 (68)	37 (66)	
				
*Treatment line—no. (%)*				
First line	23 (28)	8 (32)	15 (27)	0.56
Second line	33 (41)	8 (32)	25 (45)	
Third line or later	25 (31)	9 (36)	16 (28)	
				
*Chemotherapy regimen—no. (%)*				
FOLFIRI	27 (33)	11 (44)	16 (29)	0.16
mFOLFOX6/XELOX	25/3 (35)	4/1 (20)	23 (41)	
sLV5FU2/capecitabine	23/3 (32)	8/1 (36)	17 (30)	
				
*Intake of antihypertensive drugs—no. (%)*				
Yes	16 (20)	3 (12)	13 (23)	0.37
Calcium-channel blockers	11	1	10	
Angiotensin II receptor blockers	8	2	6	
Others	2	0	2	
				
No	65 (80)	22 (88)	43 (77)	
No. of patients requiring intervention with antihypertensive drugs during bevacizumab treatment—no. (%)	11 (14)	9 (36)	2 (4)	<0.001

Abbreviations: CEA=carcinoembryonic antigen; FOLFIRI=fluorouracil/leucovorin/irinotecan; mFOLFOX6=fluorouracil/leucovorin/oxaliplatin; sLV5FU2=fluorouracil/leucovorin; XELOX=capecitabine/oxaliplatin.
